# Suppression of YAP by DDP disrupts colon tumor progression

**DOI:** 10.3892/or.2020.7843

**Published:** 2020-11-09

**Authors:** Kun Li, Jiwei Guo, Yan Wu, Dan Jin, Hong Jiang, Chengxia Liu, Chengyong Qin

Oncol Rep 39: 2114-2126, 2018; DOI: 10.3892/or.2018.6297

Following the publication of this paper, it was drawn to the authors’ attention by an interested reader that Fig. 6D contained images featuring overlapping data, which reportedly had been derived under different experimental conditions. Subsequently, further issues of data duplication were brought to light by another interested reader concerning the above article; first, certain of the images showing colony-forming assays in Fig. 4D were strikingly similar to images that had appeared in a previous publication by the same research group, and secondly, a couple of instances of data duplication were identified among the histopathological images presented within Fig. 7D.

After having considered the various issues that have been brought to light with this paper, together with an appeal from the authors that a Corrigendum be published, the Editor of *Oncology Reports* has ruled that the article should be retracted from the publication on account of a lack of overall confidence in the presented data. Note that the authors were not in agreement that the number of errors reported and identified were sufficient to merit the retraction of the article. Additionally, the authors carefully checked the raw data and drew a conclusion that the final scientific conclusions were not affected. The Editor and the authors apologize to the readership for any inconvenience caused.

